# In Vivo Durability of Polyurethane Insulated Implantable Cardioverter Defibrillator (ICD) Leads

**DOI:** 10.3390/polym16121722

**Published:** 2024-06-17

**Authors:** Anmar Salih, Tarun Goswami

**Affiliations:** 1 Department of Biomedical, Industrial and Human Factors Engineering, Wright State University, Dayton, OH 45435, USA; tarun.goswami@wright.edu; 2 Department of Orthopedic Surgery, Sports Medicine and Rehabilitation, Miami Valley Hospital, Dayton, OH 45409, USA

**Keywords:** polyurethane, lead insulation, ICD leads, failure analysis, in vivo environment, cardiac diseases

## Abstract

The 6935M Sprint Quattro Secure S and 6947M Sprint Quattro Secure are high voltage leads designed to administer a maximum of 40 joules of energy for terminating ventricular tachycardia or ventricular fibrillation. Both leads utilize silicone insulation and a polyurethane outer coating. The inner coil is shielded with polytetrafluoroethylene (PTFE) tubing, while other conductors are enveloped in ethylene tetrafluoroethylene (ETFE), contributing to the structural integrity and functionality of these leads. Polyurethane is a preferred material for the outer insulation of cardiac leads due to its flexibility and biocompatibility, while silicone rubber ensures chemical stability within the body, minimizing inflammatory or rejection responses. Thirteen implantable cardioverter defibrillator (ICD) leads were obtained from the Wright State University Anatomical Gift Program. The as-received devices exhibited varied in vivo implantation durations ranging from less than a month to 89 months, with an average in vivo duration of 41 ± 27 months. Tests were conducted using the Test Resources Q series system, ensuring compliance with ASTM Standard D 1708-02a and ASTM Standard D 412-06a. During testing, a load was applied to the intact lead, with careful inspection for surface defects before each test. Results of load to failure, percentage elongation, percentage elongation at 5 N, ultimate tensile strength, and modulus of elasticity were calculated. The findings revealed no significant differences in these parameters across all in vivo exposure durations. The residual properties of these ICD leads demonstrated remarkable stability and performance over a wide range of in vivo exposure durations, with no statistically significant degradation or performance changes observed.

## 1. Introduction

**Figure 1:**
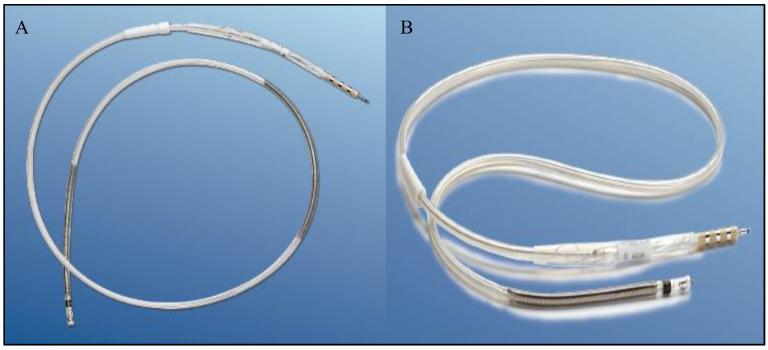
ICD leads used in the investigation: (**A**) 6947M Sprint Quattro Secure features dual coils and (**B**) 6935M Sprint Quattro Secure S with a single coil.

**Figure 2:**
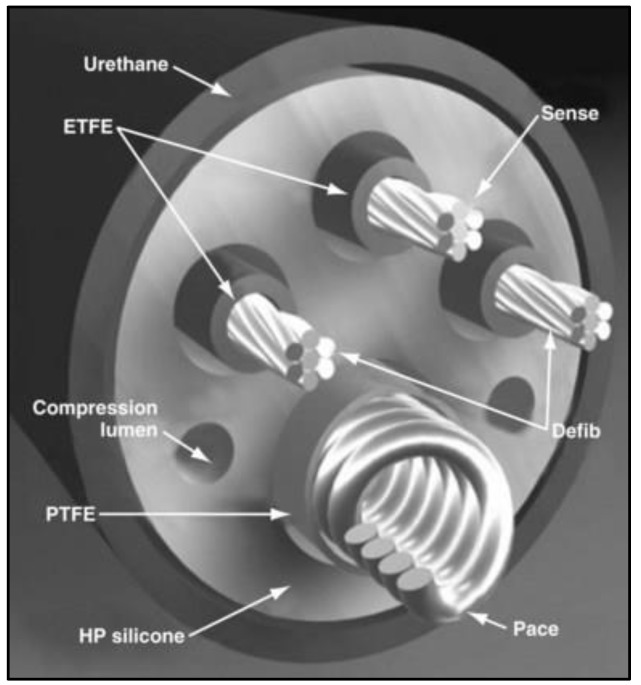
ICD lead design, featuring silicone insulation and a polyurethane outer coating. Each lumen is designated for high voltage and pace–sense conductors. The inner coil is protected by polytetrafluoroethylene (PTFE) tubing, while other conductors are encased in ethylene tetrafluoroethylene (ETFE), enhancing the structural integrity and functionality of the leads [9].

The primary challenges associated with high-voltage defibrillator leads present a significant obstacle in the scope of device therapy. More than 200,000 implantable cardioverter defibrillators (ICDs) are implanted annually worldwide, highlighting the device’s crucial role in managing life-threatening cardiac conditions. This high number reflects the growing recognition of ICDs as a standard preventative treatment for patients at significant risk of sudden cardiac arrest due to severe arrhythmias [1]. During routine implantable cardiac defibrillator (ICD) interrogations, approximately 66% of lead defects can be detected through analysis of electrical parameters. Nevertheless, in around 33% of instances, the identification of a lead defect only occurs after an inappropriate shock has already been delivered. This underscores the ongoing issue of delayed recognition of lead problems, emphasizing the need for improved monitoring and diagnostic strategies in device management [2]. An implantable cardioverter defibrillator (ICD) is a small, battery-powered medical device implanted in the chest to monitor and regulate heart rhythms. It is designed to prevent sudden cardiac arrest in patients with life-threatening arrhythmias. The ICD continuously tracks the heart’s activity, detecting irregular rhythms such as tachycardia or fibrillation. When an abnormal rhythm is identified, the device delivers an electric shock to restore a normal heartbeat [2,3]. The 6935M Sprint Quattro Secure S and 6947M Sprint Quattro Secure (Medtronic, Minneapolis, MN, USA) are high voltage leads designed to deliver a maximum of 40 joules of energy to terminate ventricular tachycardia or ventricular fibrillation. These leads play a crucial role in delivering therapeutic energy to address serious cardiac arrhythmias, providing a significant intervention in the management of these life-threatening conditions [3]. As illustrated in Figure 1, the 6935M Sprint Quattro Secure S functions as an 8.6 F (2.8 mm) active fixation true bipolar ICD lead with a single coil. In comparison, the 6947M Sprint Quattro Secure is also an 8.6 F (2.8 mm) active fixation true bipolar ICD lead but features dual coils. Figure 2 provides an insight into the design of these leads, with silicone insulation and a polyurethane outer coating for both leads. Each lumen is dedicated to accommodating high voltage and pace–sense conductors. The inner coil is shielded with polytetrafluoroethylene (PTFE) tubing, while other conductors are enveloped in ethylene tetrafluoroethylene (ETFE), contributing to the structural integrity and functionality of these leads [4]. 

The leads are situated in a challenging environment of bodily fluids; in that case, insulation materials must meet severe criteria concerning their resistance to penetration, stretchability at the point of rupture, response to blood clotting, and solid mechanical and chemical stability. Additionally, certain materials may also be needed to exhibit retention [4]. Historically, various materials such as polyamide (nylon), polyurethane, polyethylene, and silicon rubber underwent testing for lead conductors’ insulation [5]. Presently, silicon rubber and polyurethane are the predominant choices for this purpose. Silicon rubber exhibits chemical stability within the body, avoiding inflammatory or rejection responses [6]. Nevertheless, it tends to be adhesive to the touch, making the operation of two leads composed of this material within a vein challenging. This inconvenience, however, can be mitigated through the application of diverse surface coatings on the silicon insulation, enhancing the ease of handling and addressing the sticky nature associated with this material [3]. Polyurethane demonstrates notable resistance to tearing and is less prone to stickiness. Variants such as polyurethane 55D, 75D, or 90A are frequently employed, particularly for components experiencing elevated mechanical stress. However, lead insulation crafted from 80A polyurethane has proven insufficient due to microscopic cracks in the surface. Additionally, polyurethane is susceptible to degradation through metal ion oxidation, particularly when silver chlorides are present in drawn-brazed-strand conductors [7,8].

Hauser et al. [4] conducted a comprehensive investigation into internal insulation breaches (IBR) in ICD leads, specifically comparing the Durata lead (Abbott, Sylmar, CA, USA) with the Endotak Reliance (Boston Scientific, Minnetonka MN, USA) and Sprint Quattro Secure (Medtronic, Minneapolis, MN, USA) leads. The study searched into the data from the Manufacturer and User Facility Device Experience (MAUDE) database, focusing on Durata’s returned product analyses (RPA) submitted to the FDA from 2008 to 2018. The findings were then compared with RPA data from MAUDE for Sprint Quattro Secure and Endotak Reliance ICD leads. According to the study, the primary cause of Durata failure was identified as internal insulation breaches, constituting a significant 93% of failures. In contrast, Sprint Quattro Secure failures were predominantly attributed to conductor fractures, with 71% of these incidents involving the pace–sense conductor. Endotak Reliance leads exhibited an equal proportion of failures due to internal insulation breaches or conductor fracture, while 24% of malfunctions were linked to the calcification of the shocking coil and/or the pacing electrode. This comparative analysis sheds light on the distinctive failure patterns among these ICD leads, emphasizing the importance of understanding and addressing specific failure modes for improved device reliability and patient safety.

## 2. Materials and Methods

**Figure 3:**
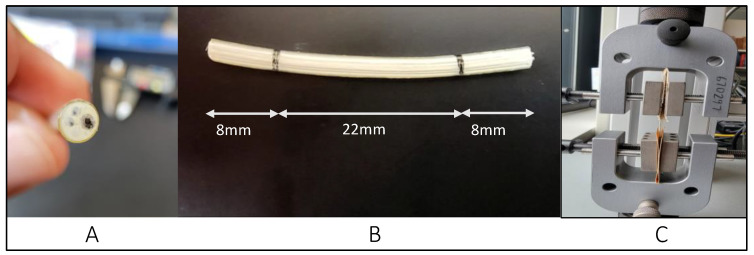
(**A**) Cross section of the lead, (**B**) length of lead and in the grip with 8 mm (**C**) during the test procedure.

We utilized thirteen ICD leads in this study. These leads are active fixation true bipolar with either single or dual coils type with a length of 52, 58, 65, 75, and 100 cm. These leads are used with silicone insulation and a polyurethane outer coating for both leads [10,11] and are manufactured by Medtronic, Minneapolis, MN, USA. The as-received devices were collected as they became available for the study by the WSU Anatomical Gift Program. The testing was conducted using the Test Resources Q series system, which was used earlier in previous studies [12,13,14]. That test applies specific displacements or loads to various samples and measures the corresponding load or displacement. The XY software on the connected computer acquires both load and displacement values, along with sampling points, ensuring precise data capture during the testing process. The in vivo exposure of the as-received leads varied for each lead, ranging from less than a month to 89 months, with an average in vivo duration of 41 ± 27 months. Age was categorized into three groups: A—younger than 65 years old, B—between 65 and 75 years old, and C—older than 75 years old. Gender classification included male and female categories. Figure 3 shows the test procedure, the testing fixture, and a cross-section of a sample under a microscope, revealing the coils and insulators. Our tests adhered to the ASTM Standard D 1708-02a [15] and the ASTM Standard D 412-06a [16].

All the tested leads adhered to a standardized sample length of 38 mm, of which 8 mm was secured within the grips, and 22 mm was positioned between them. We tested the intact left ventricle leads, including both the outer insulator (polyurethane) and the inner insulator (silicone) with the coil inside the insulation. This approach aimed to simulate the real-life conditions in which the leads would be functioning within the human body, ensuring a comprehensive evaluation of the mechanical behavior of the entire lead assembly, and to prevent any slippage during the process, the leads were firmly affixed within the grips using sandpaper. Throughout the tensile test, specific loads were systematically applied to the samples, and the corresponding displacement was meticulously measured until the insulation broke. The tensile test was repeated a minimum of two to three times, and the resultant data were averaged for reliability. Furthermore, the diameter of each specimen was measured at three distinct locations, and the average diameter was calculated (2.8 mm). A standardized gauge, with a length of 22 mm and 8 mm inside the upper and lower grips, was consistently utilized for all specimens. The tensile test itself was executed at a controlled rate of 1 mm/s, with meticulous observation of the lead’s extension. Post the separation of lead insulation, crucial parameters such as load to failure, elongation to failure, percentage elongation at 5 N, ultimate tensile strength, and the modulus of elasticity were calculated. Finally, a comparative analysis of this data was conducted, taking into consideration the in vivo exposure duration in years, gender, and age.

## 3. Results

### 3.1. Maximum Load to Failure

This study focused on assessing the maximum load required to induce failure in the outer insulation of ICD leads, considering three key factors: in vivo duration, age, and gender. In vivo duration denotes the duration the leads were exposed to the in vivo environment within the human body. Age was categorized into three groups: A—younger than 65 years old, B—between 65 and 75 years old, and C—older than 75 years old. Gender classification included male and female categories (six females and seven males).

**Figure 4:**
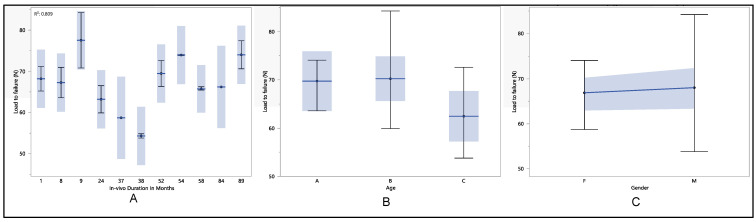
(**A**) shows the mean maximum load to failure vs. number of in vivo months of exposure, (**B**) shows the mean maximum load to failure vs. age, and (**C**) shows the mean maximum load to failure vs. gender.

As shown in Figure 4, the maximum load to failure exhibited a range of values, with a maximum of 74.3 N, a minimum of 52.1 N, a median of 66.2 N, and a mean of 65.6 N. It is noteworthy that the in vivo exposure durations varied from one month to as long as 89 months, with an average duration of 41 ± 27 months. Statistical analysis revealed no significant differences in the maximum load to failure concerning in vivo duration (*p*-value = 0.08), gender (*p*-value = 0.16), and age (*p*-value = 0.7). The maximum load to failure initially increased from 65.4 ± 5.7 N (for in vivo exposure less than a month) to 67.3 ± 5.2 N at 8 months, followed by a decline to 60.8 ± 1.4 N at 38 months. Subsequently, it exhibited fluctuations until stabilizing at 69.5 ± 4.4 N and 70.4 ± 0.5 N at 52 and 54 months, respectively. Stability persisted until 89 months when the maximum load to failure reached 71.5 ± 1.3 N. When comparing maximum load to failure with age, a decreasing trend was observed, although without statistical significance (*p*-value = 0.7). Specifically, for individuals less than 65 years old, the mean load to failure was 67 ± 4.3 N; for those between 65 and 75 years old, it was 65.6 ± 6.8 N, and for those older than 75, it was 64.3 ± 4.7 N. Furthermore, although no statistical difference was found gender-wise (*p*-value = 0.16), the maximum load to failure was higher in males compared to females. Specifically, the mean load to failure was 63.9 ± 5.7 N for females and 67.1 ± 5.2 N for males.

### 3.2. Maximum Elongation

**Figure 5:**
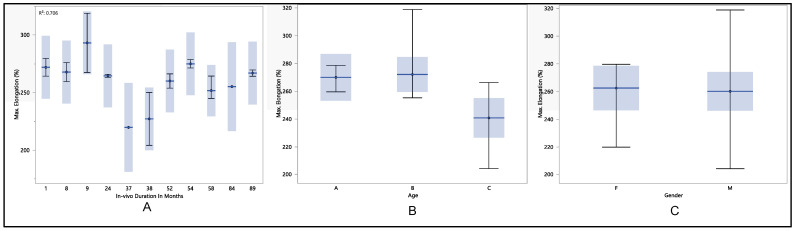
(**A**) shows the mean maximum percentage of elongation vs. number of in vivo months of exposure, (**B**) shows the mean maximum percentage of elongation vs. age, and (**C**) shows the mean maximum percentage of elongation vs. gender.

We conducted a comprehensive examination of maximum elongation, a critical parameter in our study. The mean maximum elongation was determined to be 260.8% ± 12.8%, with a minimum of 232.8%, a maximum of 279.7%, and a median of 264.3%. Our investigation considered three primary factors: the longevity of in vivo exposure measured in months, gender, and age. Statistical analysis revealed no significant differences in the maximum percentage of elongation concerning the duration of in vivo exposure (*p*-value = 0.103) or gender (*p*-value = 0.705). Although there was a decrease in maximum elongation at 37 and 38 months in the in vivo environment, this decline did not reach statistical significance. However, a significant association was observed with age group (*p*-value = 0.004). Specifically, there was a statistical difference in the age group C (>75 years old) compared to the other age groups (A < 65 years old and B between 65 and 75 years old). As illustrated in Figure 5, the maximum elongation during the first month of in vivo exposure was 265.7% ± 13.4%. Subsequently, this value increased to 269.7% ± 3.2% after nine months, maintaining stability until the 38-month mark, where it declined to 242.7% ± 10.6%. Following this decline, the maximum elongation rebounded to 275% ± 5.1% after 54 months of in vivo exposure, remaining stable until the 89-month mark, with a maximum elongation of 266.9% ± 3.8%. When examining the relationship between maximum elongation and age groups, a statistically significant decline was observed in age group C (*p*-value = 0.004). Specifically, the mean maximum elongation was 269.7% ± 7.2% for group age A, 263.4% ± 12.1% for group age B, and 248.9% ± 9.6% for group age C. Conversely, the statistical analysis indicated no significance in maximum elongation concerning gender (*p*-value = 0.705). The mean maximum elongation for females was 263.8% ± 13.4%, while for males, it was 259.8% ± 12.7%.

### 3.3. Percentage Elongation at 5 N

According to EN standard 45502-2-1 [17] and Lennerz et al. [18], the lead needs to bear at least 5 N force for one minute in order to obtain market approval. Thus, it is important to examine and study the residual properties of the insulation at this force. The maximum elongation at 5 N force was found to be 5.7%, and the minimum was 0.45%, with a median of 1.5% and a mean of 1.6% ± 1.05%. Three main factors are taken into consideration: longevity of in vivo exposure represented by number of months, gender, and age. After performing statistical analysis, it was found that there was no significant difference in the lead performance when compared to in vivo duration exposure (*p*-value = 0.15), age (*p*-value = 0.2), and gender (*p*-value = 0.33).

**Figure 6:**
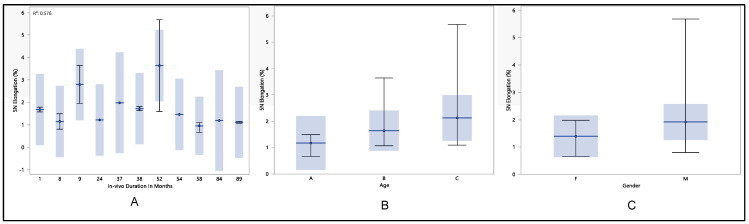
(**A**) shows the mean percentage elongation at 5 N vs. number of in vivo months of exposure, (**B**) shows the mean percentage elongation at 5 N vs. age, and (**C**) shows the mean percentage elongation at 5 N vs. gender.

As illustrated in Figure 6, the percentage elongation at 5 N exhibits a declining trend with increasing in vivo exposure duration. Despite the absence of a significant difference in elongation after 89 months, the insulation displays varying behavior across different durations. The 5 N elongation was 1.7% ± 0.1% during the initial month of in vivo exposure, fluctuating until it stabilized but subsequently declined at 54 months to reach 1.5% ± 0.0016%. This decline persisted, reaching 1.1% ± 0.05% at 89 months of in vivo exposure. Contrastingly, when evaluating 5 N elongation in relation to age groups, an increasing trend is observed. The mean 5 N elongation was 1.1% ± 0.4% for age group A, 1.6% ± 0.8% for age group B, and 2.1% ± 1.6% for age group C. Furthermore, although no significant difference was found between males and females, the 5 N elongation was higher in males compared to females, measuring 1.8% ± 1.4% and 1.4% ± 0.4%, respectively.

### 3.4. Ultimate Tensile Strength

The examination of another key property focuses on the ICD insulation leads: the ultimate tensile strength. This parameter represents the maximum stress a material can endure when stretched or pulled before experiencing necking or deformation. The analysis revealed a maximum tensile strength of 11.7 MPa, a minimum of 9.3 MPa, a median of 10.1 MPa, and a mean of 10.3 MPa ± 0.8 MPa. Statistical analysis highlighted a noteworthy decline only during the 38 months of in vivo exposure (*p*-value = 0.29), followed by a return to the previous trend until reaching 89 months, with a slight dip. Age and gender did not exhibit statistical significance, with *p*-values of 0.32 and 0.64, respectively.

**Figure 7:**
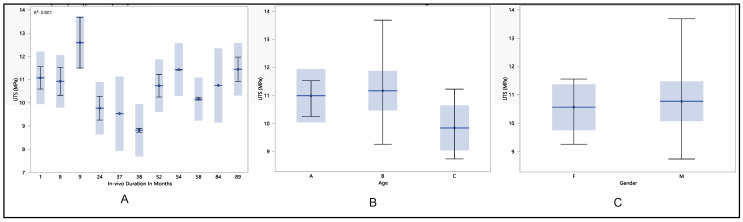
(**A**) shows the mean ultimate tensile strength vs. number of in vivo months of exposure, (**B**) shows the mean ultimate tensile strength vs. age, and (**C**) shows the mean ultimate tensile strength vs. gender.

The ultimate tensile strength commenced at 10.6 MPa ± 0.92 MPa after one month of in vivo duration, remaining stable until 37 months, when it decreased to 9.5 MPa ± 0.2 MPa. Subsequently, the ultimate tensile strength resumed its upward trend after 54 months, reaching 11.4 MPa ± 0.02 MPa, and maintained stability until 89 months, registering 10.4 MPa ± 0.75 MPa. The overall trend displayed a minor decline until the 89-month mark of in vivo exposure. Age was a significant factor in the investigation, as illustrated in Figure 7B, showing a decline in ultimate tensile strength from 10.7 MPa ± 0.9 MPa for age group A to 10.1 MPa ± 0.5 MPa for age group C. While no significant difference in ultimate tensile strength was observed between genders, there was a slight elevation in males compared to females, with values of 10.4 MPa ± 0.7 MPa and 10.2 MPa ± 0.85 MPa, respectively.

### 3.5. Modulus of Elasticity

**Figure 8:**
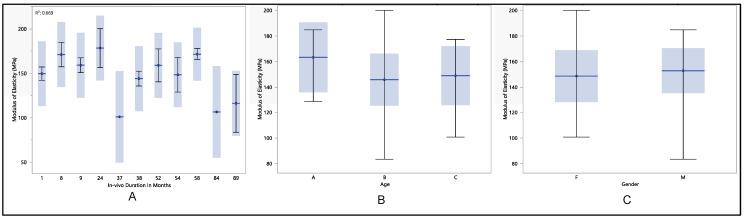
(**A**) shows the mean modulus of elasticity vs. number of in vivo months of exposure, (**B**) shows the mean modulus of elasticity vs. age, and (**C**) shows the mean modulus of elasticity vs. gender.

The research mainly focused on the modulus of elasticity as a key outcome. The study showed a maximum modulus of elasticity of 177.4 MPa, a minimum of 125.5 MPa, a median of 152.2 MPa, and a mean of 150.1 MPa ± 12.8 MPa. In-detailed statistical analysis demonstrated a lack of significant differences after 89 months (*p*-value = 0.2) across various age groups (*p*-value = 0.14) and between genders (*p*-value = 0.53). As shown in Figure 8, an obvious decline in the modulus of elasticity was observed with the increasing duration of in vivo exposure. Over the course of 9 months, the modulus of elasticity increased from 144.02 MPa ± 12.1 MPa to 161.02 MPa ± 8.9 MPa. Afterward, it kept declining to reach 143.9 MPa ± 11.7 MPa after 38 months and experienced an increase to 153.2 MPa ± 6.3 MPa after 54 months in the in vivo environment. The modulus of elasticity reached its lowest mean after 89 months of in vivo exposure, registering at 137.1.1 MPa ± 16.3 MPa. Despite the absence of significant differences between age groups, there was an evident decline in modulus of elasticity with age increasing. The modulus of elasticity was found to be 158.7 MPa ± 5.6 MPa for age group A and declined to 149.5 MPa ± 14.5 MPa for age group C. Regarding gender differences, the study identified a minor increase in modulus of elasticity in males compared to females, with values of 151.6 MPa ± 13.8 MPa and 148.4 MPa ± 12 MPa, respectively.

## 4. Discussion

The mechanical characteristics of thirteen leads were examined regarding the duration of in vivo exposure, age, and gender. Due to variations in lead length, the number of tests conducted differed for each lead, and a comprehensive examination ensured the absence of any pre-existing damage. The assessment included testing the insulation with the coil as a whole unit, which means the whole ICD leads, including both the outer insulator (polyurethane) and the inner insulator (silicone), with the coil inside the insulation. During the test, the load was applied to the intact lead. This process entailed securing the insulator’s outer wall against sandpaper and subjecting it to load during the test. Prior to individual tests, each device underwent meticulous inspection for surface defects, including those on the leads. Understanding the evolution and deterioration of residual properties over time is crucial. The degradation of materials is influenced by in vivo conditions such as temperature, moisture, and exposure to chemicals.

In this research, we introduced a mathematical predictive model in our study for load to failure, percentage of elongation to failure, percentage of elongation at 5 N, ultimate tensile stress, and modulus of elasticity. A summary of these equations is provided in Table 1. The prediction equation represents a mathematical relationship between the in vivo exposure time (τ) and the property being predicted. All of the models show a consistent trend of decreasing material properties as in vivo exposure time (τ) increases. The significance levels vary, with some models having lower *p*-values, indicating a more statistically significant relationship.

**Figure 9:**
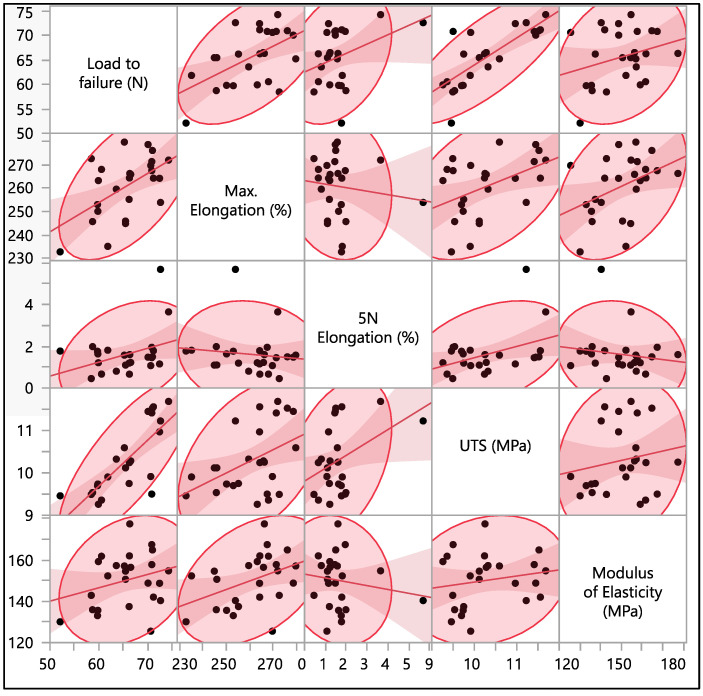
Correlation between load to failure, maximum elongation, percentage elongation at 5 N force, ultimate tensile strength, and modulus of elasticity.

Figure 9 shows the correlation coefficients between different material properties. Load to failure shows a strong positive correlation with UTS, a moderate positive correlation with maximum elongation, and a weaker positive correlation with 5 N elongation and modulus of elasticity. This implies that as a material’s load-carrying capacity increases, its overall strength, as measured by other residual properties, tends to increase as well. Maximum elongation shows a positive correlation with load to failure, UTS, and modulus of elasticity. However, it shows a negative correlation with 5 N percentage elongation. As the maximum elongation of the material increases, there is a tendency for the load required to cause failure, and ultimate tensile strength and modulus of elasticity also increase. On the other hand, the negative correlation suggests that there is a slight tendency for materials with higher elongation to have lower elongation at 5 N. This means that materials that can stretch more before failure might reach a certain elongation level at a lower force. Percentage elongation at 5 N force shows a positive correlation with load to failure, maximum elongation, and UTS, while it shows a negative correlation with a modulus of elasticity. Ultimate tensile strength shows a positive correlation with all other residual properties. This is expected; the positive correlations between UTS and other properties are in line with the expected behavior of materials. Stronger materials, as indicated by higher UTS, typically exhibit better performance in terms of load-bearing capacity and deformation characteristics. Finally, the modulus of elasticity has a weak positive correlation with load to failure, maximum elongation, and ultimate tensile strength, while a negative correlation with percentage elongation at 5 N force.

Components within polyurethanes, particularly those made of polyether, are susceptible to oxidative deterioration, including environmental stress cracking (ESC) and metal ion oxidation (MIO) [19,20,21]. Changes in these environmental factors over time could account for variations in results, patient condition, and the percentage of lead usage. The impact of temperature on material degradation within a living organism is noteworthy [22]. The fluctuations that were observed in the residual properties of the insulation performance could be due to temperature, ass temperature fluctuations within the body can impact material properties. In that case, polyurethane is designed to withstand these conditions, maintaining structural and functional integrity even in different temperature conditions [23]. The flexibility of polyurethane makes it suitable for use in leads. This flexibility allows for ease of implantation and adaptation to the dynamic environment within the body [24]. These fluctuations in mechanical properties could be due to environmental stress cracking that the polyurethane faces in vivo. It should be not neglected the role of these mechanical properties at very specific and individual regions [25] of the material which could lead significant variations in the durability of ICD devices [26].ESC refers to the process by which a material undergoes cracking or degradation when exposed to a combination of mechanical stress and specific environmental conditions [27]. Oxidative deterioration in the form of ESC can occur due to the interaction of polyurethane with bodily fluids and the physiological environment [28]. Manufacturers implement various strategies to mitigate ESC in polyurethane. This may include the use of additives and coatings that enhance resistance to environmental stress cracking [29]. Understanding the specific in vivo conditions and modifying the material composition accordingly is essential to prevent ESC in polyurethane-based cardiac devices.

Van Malderen et al. [30] conducted a study presenting the failure rates of three different leads available in the market. One crucial criterion assessed in the study is insulation breach, which has the potential to result in inappropriate therapy delivery to the patient. Insulation breach in an implantable cardioverter defibrillator (ICD) lead may be attributed to inside–out insulation abrasion, wherein the movement of internal conductors causes the insulation to deteriorate from the inside. This implies that mechanical stress and movement within the lead may contribute to the degradation of insulation, posing a risk of lead failure. The consequence of such breaches includes the possibility of internal short circuits between the right ventricular cables and shock coils. This, in turn, can lead to the occurrence of inappropriate therapy from the ICD, such as delivering shocks when they are not medically necessary. The significance of these findings underscores the potentially serious consequences associated with insulation breaches in leads, as they have the potential to result in the malfunctioning of the entire ICD system and the administration of inappropriate therapy for the patient.

In several instances, an insulation defect leads to the movement of the coil at locations subjected to significant physical stress [31]. This is typically observed as the externalization of wires, with or without associated electrical issues. Clinically, these electrical short circuits become apparent only when the outermost layer of ethylene tetrafluoroethylene (ETFE) insulation wears away, allowing direct contact between the metal components of the high-voltage (HV) circuit. The examination revealed that insulation defects represent the most significant damage score in both the proximal and distal segments of the lead, accounting for 12.82% and 28.52% of the total score in these respective regions [32]. In contrast, abrasion was identified at a rate of 10.1% in the proximal part and 5.09% in the distal part. This underscores the prominence of insulation defects in the overall assessment of lead damage, particularly in the distal portion, where the prevalence is notably higher. Abrasion can be a result of lead-to-lead contact orlead-to-can contact other structures, especially in areas where the lead experiences repetitive motion [33]. The design and flexibility of the ICD lead play a role in abrasion avoidance. Over time, exposure to physiological conditions, such as body fluids and temperature variations, may contribute to material degradation and abrasion [34,35].

Degradation of polyurethane in ICD leads can have various consequences, potentially affecting the performance and reliability of the device [35]. Degradation can weaken the mechanical integrity of the polyurethane material. This may lead to increased vulnerability to physical stress, such as bending, flexing, or compression. Mechanical failure could result in the breakage or disconnection of the lead, impacting its structural stability within the body. Polyurethane degradation can make the lead more susceptible to fracture. This is particularly concerning in regions where the lead experiences repetitive movement or bending, such as the proximal and distal parts. Premature wear and tear may necessitate early replacement of the lead, increasing the frequency of surgical interventions for patients with implanted devices. The potential consequences of polyurethane degradation pose risks to patient safety. Ensuring the reliability and integrity of ICD leads is crucial for the effective management of cardiac conditions and the overall well-being of patients.

Researchers investigated the predictive factors of lead failure in patients with cardiac devices, with a focus on identifying the clinical factors associated with the occurrence of lead failure [35]. It included 735 consecutive device implantations and found that lead failure occurred mainly with ICD leads. The study revealed that several predictive factors of lead failure were identified, including age, gender, lead insertion method, lead model, and patient diagnosis. The study showed that patients with a younger age and male gender had a higher likelihood of experiencing lead failure. These findings suggest that age and gender play a crucial role in determining the risk of lead failure in patients with implanted cardiac devices.

**Figure 10:**
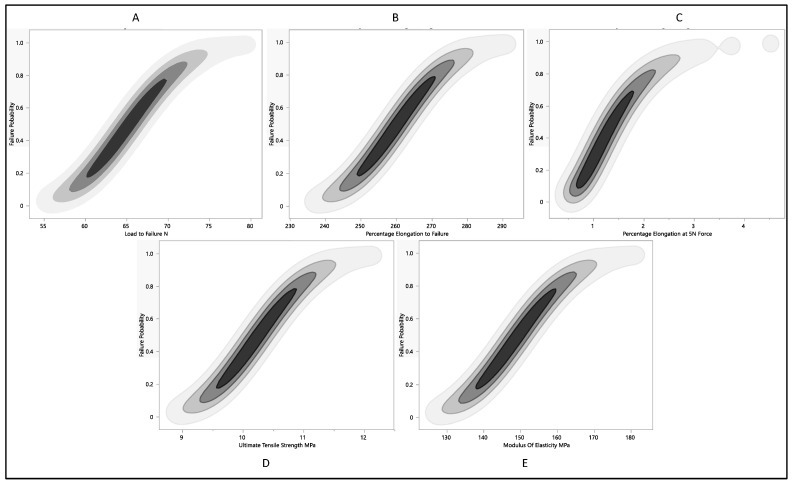
Probability of failure to each residual property with respect to (**A**) load to failure, (**B**) percentage elongation to failure, (**C**) percentage elongation at 5 N force, (**D**) ultimate tensile strength, and (**E**) modulus of elasticity.

The probability of failure represents the likelihood that the material will fail under given conditions. It is a fundamental parameter in reliability engineering, risk assessment, and safety analysis. Understanding the probability of failure is crucial for making informed decisions in engineering, design, maintenance, and operations. By examining the relationship between the probability of failure and maximum load to failure, as shown in Figure 10A, we can see that the probability of failure increases as the maximum load to failure increases. Higher failure probabilities often indicate weaker or less reliable systems or materials. The percentage elongation increases in the length of a material at the point of failure compared to its original length, as shown in Figure 10B. It is a measure of ductility or the ability of a material to deform before fracturing. We can examine how the probability of failure changes as the percentage elongation at 5 N varies, as in Figure 10C. This analysis helps understand how ductility, represented by percentage elongation, influences the likelihood of failure. Higher ductility typically indicates a greater ability to withstand deformation before failure, potentially leading to lower probabilities of failure. By studying the relationship between the probability of failure and ultimate tensile strength—Figure 10D—we can assess how material strength affects failure likelihood. Materials with higher ultimate tensile strength are generally more resistant to failure under tension. Therefore, we might expect a negative correlation between the probability of failure and ultimate tensile strength, where higher-strength materials tend to have lower probabilities of failure. Analyzing the relationship between the probability of failure and modulus of elasticity—Figure 10E—provides insights into how material stiffness influences failure probability. A higher modulus of elasticity indicates greater stiffness, meaning the material is less prone to deformation under load. Therefore, we might expect materials with higher moduli of elasticity to have lower probabilities of failure.

## 5. Conclusions

The study demonstrated a noteworthy level of stability in the residual properties of 6935M Sprint Quattro Secure S and 6947M Sprint Quattro Secure leads across a diverse range of in vivo exposure durations. Comparative analysis indicated no statistically significant differences in load to failure, percentage elongation to failure, percentage elongation at 5 N force, ultimate tensile strength, and modulus of elasticity (*p*-value = 0.08, *p*-value = 0.103, *p*-value = 0.15, *p*-value = 0.29, and *p*-value = 0.2), respectively, concerning in vivo exposure time. Analysis of the leads in relation to patient gender revealed no significant differences in load to failure, percentage elongation to failure, percentage elongation at 5 N force, ultimate tensile strength, and modulus of elasticity (*p*-value = 0.16, *p*-value = 0.705, *p*-value = 0.33, *p*-value = 0.64, and *p*-value = 0.53), respectively. Similarly, no significant differences were observed in load to failure, percentage elongation at 5 N force, ultimate tensile strength, and modulus of elasticity concerning patients’ age (*p*-value = 0.7, *p*-value = 0.2, *p*-value = 0.32, and *p*-value = 0.14). However, a significant association was observed with the age group (*p*-value = 0.004). The absence of statistically significant degradation or alterations in performance suggests the robust and reliable performance of these leads under varying physiological conditions. Despite the introduction of numerous factors through in vivo exposure, such as temperature fluctuations, moisture levels, and exposure to various chemicals within the body, the leads demonstrated stability for up to 89 months in an in vivo environment. Future research could focus on long-term monitoring of these leads in a larger patient population and involve advanced material characterization techniques to gain a deeper understanding of the mechanical and chemical properties of the polyurethane insulation over time. Comparative studies with other types of ICD leads, utilizing different insulation materials or designs, could be conducted to assess and compare in vivo durability and performance.

### Limitations

This investigation faced certain limitations. The results may be influenced by the testing conditions and procedures employed in the experiment. Variability could be introduced by differences in testing equipment, techniques, or protocols. Another limitation of this research was the absence of a broad range of in vivo durations. A more comprehensive understanding could have been achieved if additional leads with longer implant durations were available.

## Figures and Tables

**Table 1:** Mathematical predictive modeling for mechanical properties in materials testing.

Prediction Model	Prediction Equation	Significancy with Respect to In Vivo Exposure	Behavior during In Vivo Exposure
Load to failure	65.9 − 0.008τ	*p*-value = 0.0882	Decrease
Percentage elongation	266.04 − 0.12τ	*p*-value = 0.1034	Decrease
Percentage elongation at 5 N	1.82 − 0.005τ	*p*-value = 0.1514	Decrease
Ultimate tensile strength	10.5 − 0.005τ	*p*-value = 0.2997	Decrease
Modulus of Elasticity	155.3 − 0.14τ	*p*-value = 0.2008	Decrease

## Data Availability

The original contributions presented in the study are included in the article, further inquiries can be directed to the corresponding author.
